# How can we improve priority-setting for investments in health research? A case study of tuberculosis

**DOI:** 10.1186/s12961-019-0473-7

**Published:** 2019-07-19

**Authors:** Mishal S. Khan, Afifah Rahman-Shepherd, Hannah Painter, Helen Fletcher

**Affiliations:** 1 TB Centre, London School of Hygiene & Tropical Medicine, London, United Kingdom; 2 Centre on Global Health Security, Chatham House, 10 St James’s Square, London, United Kingdom

**Keywords:** Health research and development, prioritisation, policy

## Abstract

**Background:**

Although enhanced priority-setting for investments in health research for development is essential to tackling inequalities in global health, there is a lack of consensus on an optimal priority-setting process. In light of the current surge in tuberculosis (TB) research investment, we use TB as a case study.

**Methods:**

We investigated two critical aspects of a research prioritisation process, namely the criteria that should be used to rank alternative research options and which stakeholders should be involved in priority-setting. We conducted semi-structured interviews with 24 key informants purposively selected from four distinct groups – academia, funding bodies, international policy or technical agencies, and national disease control programmes. Interview transcripts were analysed verbatim using a framework approach. We also performed a systematic analysis of seven diverse TB research prioritisation processes.

**Results:**

There was consensus that well-defined and transparent criteria for assessing research options need to be agreed at the outset of any prioritisation process. It was recommended that criteria should select for research that is likely to have the greatest public health impact in affected countries rather than research that mainly fills scientific knowledge gaps. Some interviewees expressed strong views about the need – and reluctance – to make politically challenging decisions that place some research areas at a lower priority for funding. The importance of taking input from stakeholders from countries with high disease burden was emphasised; such stakeholders were notably absent from the majority of prioritisation processes we analysed.

**Conclusions:**

This study indicated two critical areas for improvement of research prioritisation processes such that inequalities in health are better addressed – the need to deprioritise some research areas to generate a specific and meaningful list for investment, and greater involvement of experts working in high disease-burden countries.

## Background

Every year, billions of dollars are invested in health research and development, including new or improved health products and processes [1, 2]. However, only a small proportion of these funds addresses the health issues that cause the greatest burden of disease, thereby falling into the category of health research for development (R4D) [3–5]. Thus, although investments in health research have strengthened the evidence base to improve policy setting and practice, health gains have not been distributed equitably across population groups [6]. The longstanding mismatch between health research that is necessary to improve health in settings with the greatest need, and that which funders invest in was first highlighted in 1990 by the Commission on Health Research for Development [5] – findings about the very small proportion of global health research expenditure directed toward diseases that have a large impact on mortality in low- and middle-income countries became known as the 90/10 gap [7]. Despite awareness of this issue, targeting research funding toward the health areas with the greatest need for investment remains a critical challenge [8].

Enhanced priority-setting for health R4D was therefore identified by WHO and others as an essential part of the solution to the persistent inequalities in global health improvements [3, 9]. Priority-setting aims to increase collaboration, coordination and overall investment, directing funding toward research that will have maximum benefit to society [10]. While there is a clear need to improve the process of R4D priority-setting, the definition of an optimal process remains unclear. A number of tools have been developed to address the gap and establish guidelines on research priority-setting, including a module on deliberative priority-setting developed at the Canadian Institutes of Health Research [12, 13]. However, an analysis of research priority-setting exercises undertaken by WHO found that there is no gold standard approach and that there is demand from policy-makers for normative work in this area [11]. For example, while guidelines suggest that selecting who to involve in determining priorities should be “*inclusive*”, “*transparent*” and “*strive for appropriate representation of different expertise and for balanced gender and regional participation*”, guidelines are not clear about the participation of stakeholders from for-profit organisations, high disease burden countries and sectors outside health [14]. Similarly, the values or criteria that should be used to assess and prioritise research areas are not agreed across the range of groups conducting research prioritisation since these can be subjective.

Tuberculosis (TB) provides an ideal case study owing to the large number of stakeholders that have used alternative processes for TB research prioritisation [15], and the surge in political and financial attention for TB research following the first ever United Nations General Assembly meeting on TB and the global Moscow declaration to increase investment in TB [16, 17]. In recent months, US$ 2 billion has been committed annually to TB research. However, there has been criticism of how effectively TB research money has been spent in the past [18, 19] and effective ways of prioritising allocation of this increased investment in TB research must be identified urgently [16].

Our study’s first objective was to investigate stakeholder opinion on what an ideal R4D prioritisation process might look like from diverse perspectives. In particular, we focus on two contentious areas, namely criteria that should be used to rank alternative research priorities and which expert groups should be involved in priority-setting decisions. The second objective was to assess practices followed during existing TB R4D priority processes in light of stakeholder opinions and guidelines for prioritisation.

## Methods

### Study design

Based on Viergever et al.’s nine-item checklist for priority-setting [14], we identified components of the R4D prioritisation process that were inherently subjective and considered by the WHO team responsible for setting up the Global Observatory on Health Research and Development as being those for which normative guidance was critical. We focused on two key components that can have a major impact on the outcome of the priority-setting process – (1) which criteria should be used to assess and compare alternative research options for investment, and (2) which groups of experts should be engaged as part of the prioritisation process.

We conducted a mixed methods study involving semi-structured interviews and systematic analysis of seven diverse TB R4D prioritisation processes.

### Interviews

We designed a semi-structured topic guide to investigate key informant perspectives on how an optimal R4D prioritisation process could be structured, focusing on the two key questions detailed above. Twenty-four informants were purposively selected to represent four distinct groups whose views we sought to examine and compare, namely academia (*n* = 9), funding bodies (*n* = 5), international policy or technical agencies (*n* = 6), and TB disease control programmes in low- and middle-income countries (*n* = 4; Afghanistan, Botswana, Pakistan and South Africa). Purposive selection was applied to ensure that we covered a range of geographies, academic disciplines and sectors (non-profit, for-profit) to capture diverse perspectives on health R4D.

Semi-structured interviews were conducted by members of the author team (all with MSc or PhD level qualifications) with each of the informants in person or via Skype. All authors had jointly designed the topic guide and had expertise in TB. Interviews lasted between 20 and 45 min and were voice recorded with consent in all cases, except for two informants who preferred to provide their thoughts as notes following the discussion. Detailed notes were taken during the interviews.

All interviews were anonymised and assigned individual codes. Two of the researchers, one of whom was not involved in conducting the interview, were responsible for independently transcribing voice recorded interviews verbatim. The framework method was used to facilitate the manual analysis of transcripts and notes [19]. The analysis began with an initial round of deductive coding by a third researcher not involved in the transcription process to minimise bias related to the researchers own TB research interests, which included epidemiology, policy research and immunology. This was followed by a round of inductive coding by all the authors to allow recurring themes to be identified. Attention was paid to differences and similarities between views expressed by different informants and we noted that data saturation was reached with respect to views on the two questions of focus outlined in the introduction.

### Systematic analysis of existing TB R4D prioritisation processes

To assess current practices with respect to R4D prioritisation and compare these with key informant views on this, we analysed seven diverse R4D prioritisation processes in terms of the criteria used to assess research options and the stakeholders involved. We used a two-step method to identify TB R4D prioritisation processes for comparison. In the first step, the authors screened peer-reviewed and grey literature, and conducted an online consultation with members of the London School of Hygiene and Tropical Medicine TB Centre, the WHO team responsible for setting up the Global Observatory on Health Research and Development, and the WHO Global TB Programme. Based on the initial set of TB R4D processes identified, we contacted leaders of each R4D process to collect further information about their system and to provide details about any other tools they were aware of, using a snowballing methodology.

We identified 14 unique R4D prioritisation tools or processes and included seven in our analysis that had been used to prioritise between TB research investment options, with or without consideration of other diseases in addition to TB (Table 1). Processes that generated published recommendations and those conducted by funding agencies that did not generate publicly available recommendations were included (for the latter, we contacted the organisation responsible for conducting the process to collect relevant data, and de-identified data before presenting it to ensure the confidential information was not shared outside the organisation).

**Table 1:** Research for development prioritisation processes analysed

Name of process	Owner
Internal Prioritization Assessment	Private for-profit
Special Programme for Research and Training in Tropical Diseases	WHO
International Roadmap for TB Research	WHO
Priorities for TB Research: A Systematic Review	Study authors
TB Program Strategy	Private non-profit
Vaccine Research and Development Prioritisation	Private non-profit
Research and Development Funding Gaps Analysis	Government research agency

Information about the criteria used to assess research options and the stakeholders involved in each R4D prioritisation process was extracted from published reports or scientific papers, or by means of a structured discussion with individuals responsible for leading the process of interest, using a standardised data collection tool. Information was captured in a standardised data capture form to ensure that all questions were covered adequately, and the use of multiple-choice answers facilitated standardised data collection across the seven R4D prioritisation processes. Definitions used to classify the different stakeholder groups and to classify the criteria used for comparison during our analysis are presented in Table 2. The same researcher extracted data about all processes to ensure that a standardised approach was applied, and at least one other researcher checked the data extraction as a quality control measure.

**Table 2:** Standardised tool used to collect information about the processes

Tool component	Standard definition used to determine which stakeholders and criteria were relevant
Which stakeholders were involved as experts? [tick all that apply]	• Academics o From a single discipline such as one of: basic science, epidemiology, operational/translational research, health economics, health policy and systems research, etc. o From multiple disciplines: two or more disciplines • International policy-makers/technical experts: representatives of WHO and other policy or technical assistance bodies working across multiple countries • National disease control programme representatives • Civil society: advocacy groups, community groups, etc. • Funding body representatives: Funding Gaps Analysis, TB Program Strategy, Wellcome Trust, etc. • Patients • Physicians: whose primary occupation is treating (TB) patients in high-burden settings • Industry representatives or product development partnerships
What criteria/values were used to prioritise areas? [tick all that apply]	• Effectiveness/efficacy: impact on reducing disease burden or adverse consequences of disease • Knowledge gap: addressing critical scientific knowledge gaps that limit progress on disease control • Cost-effectiveness: cost of delivery relative to impact is appropriate for high disease-burden settings • Deliverability: investment will produce an output that can be implemented and deliver impact in settings with high disease burden (feasibility) • Equity: knowledge or tool produced will benefit all populations, including vulnerable groups and populations in low-resource settings • Sustainability: output implementation can be supported by finances and infrastructure available in high disease burden settings for the long term • Other (specified)

## Results

Overall, there was consensus among informants from all four groups that the purpose of the prioritisation exercise and, linked to this, the criteria used to assess research options need to be clearly defined at the outset. Informants believed that the remaining steps in the process, including which stakeholders are involved in making prioritisation decisions and what information should be presented to them, will follow directly from these first decisions. However, informants recognised that making decisions about which criteria to use and which stakeholders to involve can be highly political, and will depend on the values and strategic objectives of the organisation leading the prioritisation process (A07).

Our detailed findings are structured according to the two components of the R4D prioritisation process that we focused on.

### Selecting criteria to assess alternative R4D options

There was a strong sentiment, particularly among funding body representatives, that the criteria or values used to prioritise R4D options for investment are neither clear nor specific enough (F02, F04, F05, A07). These informants thought that the purpose – for example, to maximise benefit to the affected populations or, alternatively, to reduce inequalities in health – should be linked to clearly articulated criteria that act as the basis for prioritisation decisions. However, it was also recognised that it is politically challenging for an organisation, such as WHO, to take a strong position on which criteria (and therefore which research areas) are most important, for example, deciding between cost-effectiveness and equity impact of research investments, which are at times mutually exclusive.

Elaborating on this point, a funding agency employee (F05) asserted that research prioritisation lists published by WHO are less useful than they could be, possibly doing more harm than good in terms of attracting interest from funding bodies because they appear to capture everything as a priority, without sufficient narrowing down or de-prioritisation.

“*I’ve seen recently enough WHO people make intents to do something like…a global TB research agenda. Then they come up and say it’s going to cost US$ 4 billion, and to me that’s absolutely not helpful, that’s not helpful at all. There's something about boiling the ocean about it, when I’m hearing… a certain project could cost US$ 25 million or US$ 100 million, yes we can talk about this. When it’s US$ 6 billion, then … there's nothing I can do about that and it scares people away.. I think it actually backfires. So I don't believe in grand schemes to be honest...*” (F04)

In terms of the number of criteria that should be used, all informants were comfortable selecting three or four criteria on the basis of which they believed R4D options should be ranked. One international policy-maker (IP03), with extensive experience of prioritisation exercises, stressed that multiple criteria (three to five) should be adopted to rank options as one criterion is not sufficient. On the other hand, it was consistently recognised that too many criteria make the processes overly lengthy and complicated for participants, and that important conversations about criteria and values might be difficult as trade-offs need to be made, including de-prioritising some research areas owing to a lack of resources.

Effectiveness/efficacy and deliverability at scale within high disease-burden settings were the two criteria considered most important by the majority of informants, who reasoned that these two criteria contribute to the impact of research, which they perceived to be the key aim of investments. Several of the academics interviewed felt that impact on disease control was more important than simply addressing knowledge gaps, or minimising costs, or sustainability (A02, A03, A05).

“*I think effectiveness is certainly the most important, there’s no point having something that is deliverable and equitable and sustainable if it’s not effective, so I think the entry point is having something that is effective… Knowledge gaps, less so, less so, I think it’s primarily about identifying things that are likely to have a big impact, and working out whether or not you can deliver them at scale, and the cost will eventually take care of itself if it’s a good enough intervention.*” (A02)

Academics differed in opinion on the meaning of effectiveness, with one defining it in terms of lives saved over a 15-year period (A07) and another stressing that effectiveness should be thought of in terms of impact on the quality of life of affected people and not just in terms of deaths or new cases averted (A01). Other academics (A04, A09) and a funding agency employee (F05), however, considered cost-effectiveness to be the most important criterion for prioritising R4D options. In contrast, some programme managers from low- and middle-income countries (NP01, NP02) felt that sustainability, in terms of the availability of resources locally to implement any new intervention that is developed, is critical along with effectiveness:

“*I think for programmes, sustainability is very important, because what will happen when donors will go away? We are dependent 95% on donors now, so what will happen in future…and then obviously effectiveness is very important. Equity, yes, but I would still rank sustainability and effectiveness on top*.” (NP02)

A number of informants from different groups emphasised that deliverability needs to be assessed early, or in parallel, with effectiveness (IP01, NP02). There was recognition that, in the past, considerations about deliverability in resource-constrained health systems have been left until too late.

“*You’ve got to do your efficacy work, but don’t wait 4 years… let’s also run parallel effectiveness studies,* [and] *deliverability scale up, let’s try and get that evidence working together. I’m not sure people think like that*.” (IP01)

Related to this point, another frequent suggestion from the academics, funding agency employees and international policy-makers was that evaluation of past investments to assess real impact achieved (as opposed to projected impact) is critical to consider, although such information is rarely provided to inform prioritisation decisions.

“*We are interested in whatever we have done, how much is (achieving) good impact.*” (NP02)

Our analysis indicated that a perceived advantage of assessing actual impact retrospectively is that health systems constraints in delivery are accounted for:

“*But I would want that exercise to be grounded in reality, so to take the data we have now for TB or HIV resource tracking and start from looking historically at the investments and what they yielded and try to come up with some kind of relationship there, rather than think about it prospectively, which is too subject to vague expert opinion*.” (IP07)

**Fig. 1:**
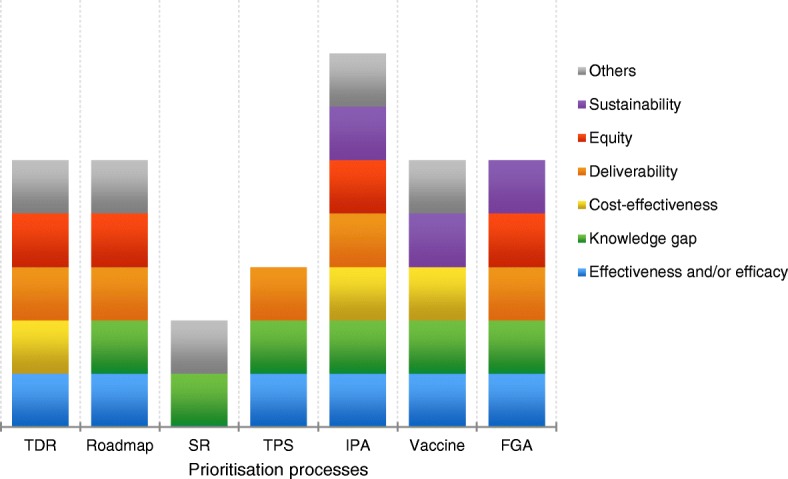
What criteria were used to prioritise? *TDR* Special Programme for Research and Training in Tropical Diseases; *Roadmap* International Roadmap for TB Research; *SR* Priorities for TB Research: A Systematic Review; *TPS* TB Program Strategy; IPA Internal Prioritization Assessment; *Vaccine* Vaccine Research and Development Prioritisation; *FGA* Research and Development Funding Gaps Analysis

Our analysis of how a range of prioritisation processes have been run showed that the majority (5/7) assessed research options against five or more criteria (Fig. 1). The ‘Internal Prioritization Assessment’ process included the highest number of criteria, while the ‘Priorities for TB Research: A Systematic Review’ and ‘TB Program Strategy’ processes included the lowest, two and three, respectively. In keeping with its purpose, the ‘Priorities for TB Research: a Systematic Review’ prioritised research options using knowledge gaps identified by the researchers as a key criterion, and additionally considered research priority areas highlighted in other publications. The most frequently used criteria among the prioritisation processes were effectiveness and/or efficacy (6/7) – which is consistent with key informant opinions – and knowledge gaps (6/7), followed by deliverability (5/7), equity (4/7), cost-effectiveness and sustainability (3/7). Additional criteria used in individual processes led by commercial or non-profit bodies with their own funding included manufacturability (Internal Prioritization Assessment) or business case (Vaccine Research and Development Prioritisation). The two processes run by groups within WHO – the Special Programme for Research and Training in Tropical Diseases and the International Roadmap for TB Research (Roadmap) – additionally considered alignment of alternative research areas to global goals (such as the Sustainable Development Goals), and whether research would bring about health gains in an ethical way while minimising potential harm to patients.

### Experts to be engaged in research priority-setting exercises

A common theme emerging from interviews was that engaging a broad range of stakeholders in prioritisation exercises is essential, with several informants (12/24) emphasising that it is critical to include stakeholders working on the ground in high disease burden countries. Stakeholders with ‘in-country’ or ‘front-line’ expertise who were identified as important to involve included national programme representatives, academics or researchers (disease specific and non-specific), and programme implementers. One government (health) programme manager and lead researcher (NP02) held the view that research officers in national programmes, as well as the managers, should be involved as they often have greater insight into prioritising research areas. In line with these suggestions, one funding agency employee felt strongly that prioritisation processes should avoid involving a small group of experts exclusively and repeatedly, which makes the process “*incestuous*” (F04). A second funding agency employee illustrated the pitfalls of failing to include those stakeholders who will ultimately be responsible for using any new tools or interventions developed in relation to TB control:

“*…to what degree do we really engage the ultimate demand stakeholders, which are, at the end of the day, the patient, the healthcare workers and the country decision-makers in this kind of development of future interventions? My gut feeling is that the lion’s share of the conversation is what can we do, from our analysis that we think would be helpful, and I don’t think we do enough of a job balancing that with a deep conversation with the country’s national TB control coordinators themselves…*” (F05)

There were mixed views about whether funding body representatives, particularly those from industry, should be involved in any ranking or voting process. Funders’ involvement for buy-in was considered important by a range of informants (NP02, IP07, IP08, F11, A03, A07, A08), but some academics (A05, A06, A09) raised concerns about involving companies that may have a commercial interest in specific research options being prioritised:

“*..so I guess I’d be quite happy for impartial research funders to be there, but maybe research funders linked to industry, I would have to be cautious about, and I guess it raises a broader question, which I’ve also hinted at before, the process whereby stakeholders are selected and conflicts of interest are put on the table and managed, I think that needs to be part of that process, it’s not just about who but how, how they’re engaged*.” (A09)

When probed about the appropriateness of them being represented in priority-setting, some funding agency employees and industry representatives commented that excluding them from processes owing to conflicts of interest is unwarranted since academics and other stakeholders may also have personal preferences that shape their views.

There were also diverging views on the involvement of civil society representatives and patients. While the importance of involving affected people was generally agreed upon, there were questions about how to best represent patients (A07).

**Fig. 2:**
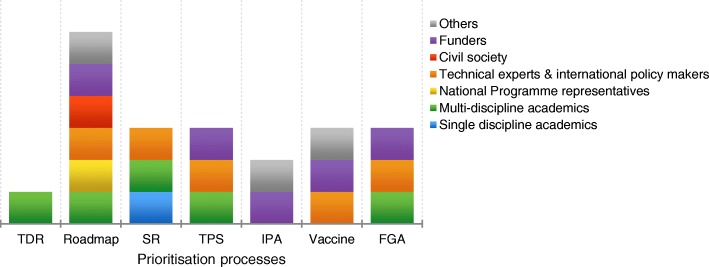
Who was involved in priority-setting?

Systematically comparing the number and types of stakeholder groups involved in determining priorities in each of the seven processes revealed substantial variations in practice (Fig. 2). Academics (multiple disciplines), funders and technical experts/international policy-makers were most frequently involved; five of the seven processes engaged representatives from these stakeholder groups. In stark contrast to the strongest recommendation from key informants, only one of the seven processes (Roadmap) involved representatives from National Disease Control Programmes and civil society groups. The Roadmap prioritisation process led by WHO engaged the highest number of stakeholders to decide on priorities, excluding only single discipline academics. However, the Special Programme for Research and Training in Tropical Diseases process, also led by a group within WHO, only engaged academics (multi-discipline) to advise on priorities, which was the lowest number of stakeholders among the compared processes. None of the processes involved TB patients or physicians treating TB.

## Discussion

Commitment to operationalise and strengthen R4D prioritisation processes has been expressed at the highest levels, most recently at the 2016 World Health Assembly [20]. A critical barrier to this has been the lack of consensus and normative research on two critical components of a R4D prioritisation process – which criteria should be applied to prioritise between alternative research options and which groups of experts should be engaged [9]. Our study, which is the first to address these questions through a qualitative investigation of views from diverse informants and a systematic analysis of practices adopted in a range of existing TB R4D prioritisation processes, revealed that R4D prioritisation processes could be improved by involving stakeholders with expertise of high disease burden settings; only one of the processes analysed involved TB programme staff from affected countries and none included frontline healthcare providers. In terms of criteria used for prioritisation, the majority of processes included effectiveness (for impact on TB), which was a key criterion suggested by the majority of informants. However, we found that R4D prioritisation processes typically assessed research areas in terms of the extent to which they addressed a scientific knowledge gap, and this was not perceived to be as important by informants.

Irrespective of which criteria are chosen, trade-offs between different criteria and politically challenging decisions on the values of most importance to the WHO (or other priority-setting agency) were seen as being essential in order to generate a more specific and meaningful list of research priorities. There were strong views among some funding agency employees in particular that failure to do this has resulted in prioritisation processes producing research investment lists that cover too many research options, presenting unrealistically large budgets for funders to respond to.

In relation to the question about which stakeholders should be engaged as part of the prioritisation process, it was recognised by several informants that all stakeholders involved in an R4D prioritisation exercise (including academics) may have some degree of bias or personal interest in supporting specific research areas, and that an unbiased ranking of R4D options may not be possible or even useful. Indeed, in line with the views of informants interviewed, studies have shown that different stakeholder groups prioritise research options differently [21, 22]. In light of the variation in stakeholder perspectives, a specific recommendation emerging was to ensure inclusion of stakeholders working at the policy or service delivery level in countries with a high disease burden. However, our analysis of prioritisation processes showed that academics and funders were represented commonly, whereas voices from policy-makers and practitioners in affected countries were not, and a potential danger of excluding these groups, raised by informants, was that the prioritisation process may result in the deliverability and acceptability of research options being less thoroughly considered. Furthermore, engagement of policy-makers and users of research in shaping and conducting the studies that are funded is closely related to action of research findings, as demonstrated in a study by Kok et al. [23]. In line with this concern, a recent analysis found that TB research conducted in Cambodia aligns poorly with domestic policy-makers’ priorities [24], and our findings may reflect a wider issue related to global agendas for health research being poorly aligned with local needs [25]. Although the recommendation to increase the number of stakeholders involved in prioritisation processes appears straightforward, we acknowledge the challenges in finding a balance between productivity (agreeing on a relatively small list of research priorities in the time available) and inclusion (including all voices and perspectives).

A limitation of our study is that we only analysed the views of a limited number of key informants, and we acknowledge that our scope excluded views of groups such as patients, healthcare providers, health system administrators and politicians. To investigate an important recurring theme about the impact of R4D prioritisation processes on shaping investments in research being limited because research lists produced are too broad or because prioritisation processes do not sufficiently take health systems constraints into account, we suggest objectively evaluating the impact of research prioritisation exercises. With increasingly more investment in improved, transparent processes and data for research prioritisation, it is important to evaluate whether prioritisation exercises have resulted in changes to investment in research areas in line with recommendations [26]. Since the majority of grants awarded to undertake research within prioritised research areas (for example, TB case finding) are allocated following a process of peer-review and rating by academics, it may be that academics end up acting as gatekeepers that can ultimately shift funding towards studies that primarily address knowledge gaps rather than those that generate findings with practical utility for affected populations. Several global health research priority-setting exercises have been conducted since the late 1990s and an assessment of their impact would indicate whether these are useful to funders and implementers [26–28]. Assessing impact of research prioritisation exercises is not straightforward, and the first step would involve defining appropriate indicators. Suggested indicators, such as changes in research output and in funding flows toward research based on the established research priorities, would be useful but complicated to track and compare across countries [11, 29]. The indicators would also stop short of assessing impact on health, since they focus on whether research priorities have changed, but not whether the prioritisation exercises result in changes to how funds are allocated to research.

## Conclusions

Decisions on prioritisation criteria and expert engagement are critical not only to the R4D prioritisation processes outcomes in terms of a recommended research priority list, but also to indicate how useful and widely accepted the recommendations are. We highlight three important findings emerging from this study that may help to inform development of enhanced R4D prioritisation processes for TB and other diseases. Firstly, although it may be politically challenging, some research areas must be deprioritised in order to generate useful recommendations from a prioritisation exercise, and this should be done on the basis of multiple, clearly articulated criteria for comparing R4D options. Secondly, there should be more attention to the health systems in which research outputs are to be implemented as part of prioritisation exercises. This may be achieved by increased involvement of policy-makers and implementers from high disease-burden countries, which is currently lacking. Finally, it should be recognised that no group of experts – including funders and academics – are entirely unbiased in their assessments of research options; all could potentially have vested interests in the outcome of the prioritisation exercise.

## Data Availability

The data are freely available from the WHO website at goo.gl/MR9DbN.

## Abbreviations

R4D: research for development
Roadmap: International Roadmap for TB Research
TB: tuberculosis
TDR: Special Programme for Research and Training in Tropical Diseases
TPS: TB Program Strategy
